# A narrative review on the effect of economic downturns on the nursing labour market: implications for policy and planning

**DOI:** 10.1186/1478-4491-10-23

**Published:** 2012-08-20

**Authors:** Mohamad Alameddine, Andrea Baumann, Audrey Laporte, Raisa Deber

**Affiliations:** 1 Faculty of Health Sciences, Department of Health Management and Policy, American University of Beirut, Riad El-Solh, Beirut, 1107 2020, Lebanon; 2 Faculty of Health Sciences International Health, Michael G. DeGroote Centre for Learning, McMaster University, Hamilton, ON, L8S 4K1, Canada; 3 Faculty of Medicine, Institute of Health Policy Management and Evaluation, University of Toronto, Health Sciences Building, 155 College Street, Suite 425, Toronto, ON, M5T 3M6, Canada

**Keywords:** Economic downturn, Nurses, Recession, Supply, Demand, Policy

## Abstract

Economic downturns and recession lead to budget cuts and service reductions in the healthcare sector which often precipitate layoffs and hiring freezes. Nurses, being the largest professional group in healthcare, are strongly affected by cost reductions. Economic downturns destabilize the nursing labour market with potential negative outcomes, including serious shortages, extending beyond the recessionary period. The objectives of this manuscript are to provide an overview of the potential short- and long-run impact of economic downturns on the supply and demand of nurses, and present healthcare decision makers with a framework to enhance their ability to strategically manage their human resources through economic cycles.

A narrative review of the literature on the effects of economic downturns on the nursing labour market in developed countries was carried out with a special focus on studies offering a longitudinal examination of labour force trends. Analysis indicates that economic downturns limit the ability of public payers and institutions to finance their existing health workforce. As salaried healthcare workers, nurses are especially susceptible to institutional budget cuts. In the short run, economic downturns may temporarily reduce the demand for and increase the supply of nurses, thereby influencing nursing wages and turnover rates. These effects may destabilise the nursing labour market in the long run. After economic downturns, the market would quickly display the pre-recessionary trends and there may be serious demand–supply imbalances resulting in severe shortages. Potential long-term effects of recession on the nursing labour market may include a downsized active workforce, difficulty in retaining younger nurses, a decreased supply of nurses and workforce casualisation.

Lack of understanding of labour market dynamics and trends might mislead policy makers into making misinformed workforce downsizing decisions that are often difficult and expensive to reverse. In the aftermath of an economic downturn, the costs of attracting nurses back often outweigh the short term cost savings. Effective management should support the nursing workforce by creating attractive and stable work environments to retain nurses at a manageable cost.

## Preface

Economic downturns affect all sectors of the economy including healthcare [1-3]. They may escalate into a recession, defined broadly as a self-reinforcing reduction in economic activity characterized by persistent job losses precipitating increased unemployment [4]. Many countries around the globe are currently in the midst of economic downturns, which are anticipated to grow into a global recession [5].

Nurses, being the largest professional group in the healthcare sector, are strongly affected by budget balancing attempts. For many institutions, the easiest and fastest means to balance the books is to cut back the nursing workforce as institutions restructure, downsize, merge or actively shift care from hospital to community [6,7]. Such restructuring may precipitate detrimental and difficult to reverse consequences in the longer run [8-10].

Now that the economy of many countries around the globe is undergoing turbulent times, it is important that the recessionary trends in the healthcare sector do not drive policy and decision makers to take decisions that may destabilise the nursing labour market for years to come. In this manuscript, we attempt to examine the effects of economic downturns and recession on the supply and demand of nurses and provide a framework to help healthcare decision makers understand and manage their valuable nursing resources during tough economic times.

### The supply and demand for nurses: a brief overview

**Figure 1:**
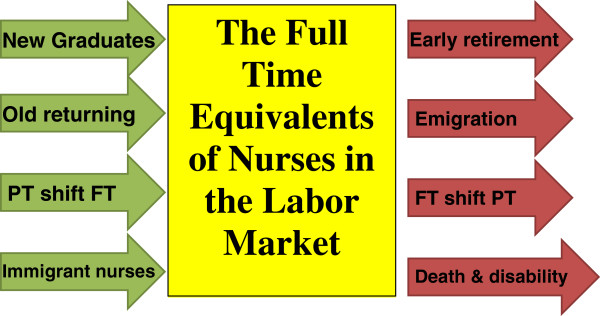
A Stock and Flow Model for the supply of nurses into the labour market.

Projecting nurses’ supply in a labour market traditionally uses a stock and flow model [11] (Figure 1). This model measures total workers’ involvement in a labour market using full-time equivalents (FTE). One FTE indicates that a worker is putting the number of work hours of a full-time worker.

In a stable fiscal environment, the total number of nurse FTEs in a labour market is replenished through various sources. These include graduates of nursing training programmes, inactive nurses returning into the labour market, recruitment of foreign-trained nurses (immigrant) into the labour market and an increased proportion of part-time/casual nurses enhancing their labour force participation by increasing the total number of hours worked. Conversely, nurse FTEs are subtracted from the labour market through death, retirement, emigration, disability and decreased labour force participation (shifting from full-time to part-time employment). Policy makers use policy levers to project and optimise the number of nursing FTEs required to address current and future anticipated needs of the population. This optimisation is a complex task compounded by many issues, such as the lag time needed to train specialised human resources and the importance of maintaining surge capacity to deal with unexpected emergencies (e.g., emerging diseases and global epidemics) [12,13].

On the demand side of the nursing labour market there is an array of potential employers (e.g., hospitals, long-term care facilities, homecare, etc.) competing for the employment of nurses with various specialties and skills. The demand for nurses thus depends on their productivity in different tasks, their skills and the degree to which they could be replaced by other employees.

## Review

In order to review and analyse the effect of economic downturns on the nursing workforce a combination of a narrative review and expert opinion were utilised. The two methods were combined towards the development of an analytical framework that could guide the human resource decisions of health decision makers and stakeholders in tough economic times.

Narrative reviews are invaluable in theory development/evaluation, problem identification or in surveying the state of knowledge on a particular topic [14,15]. A narrative review of published manuscripts on the effect of economic downturns and recession on the nursing labour market was conducted using PubMed, the Cumulative Index to Nursing and Allied Health Literature (CINAHL) and the Institute for Scientific Information (ISI) library databases. A special focus was placed on studies offering a longitudinal examination of labour force trends in developed countries. Key words used to search the title and abstract included various combinations of: recession, economic downturn and nurse(s), nursing and labor/labour market, workforce trends and employment. The search was not meant to be exhaustive but rather purposeful to help shape a clearer understanding of the effect of economic downturns on the nursing labour market.

Although the search was not limited by year of publication, the focus was on the most recent economic downturn beginning in 2007–2008 in many developed countries around the globe. The original search yielded a large number of articles that were reduced to 42 unique manuscripts after a review of the abstract for relevance. The 42 manuscripts were fully reviewed by two members of the research team to judge their type, general focus and the means through which they addressed the question of interest. After this review, nineteen manuscripts were excluded from the analysis because their main focus was not on the analysis of economic downturns, workforce trends or because they did not focus on the nursing workforce. The remaining 23 manuscripts were thoroughly reviewed by two members of the research team. Each member individually read all the articles and identified main themes relating to the effect of economic downturns on the nursing workforce. The two reviewers met and consolidated the identified themes into five main themes (demand, supply, salary, turnover and shortages) in order to enhance the clarity of analysis. The five themes were reviewed and approved by all authors and are reported in this manuscript, with reference to the articles when necessary.

This search was supplemented by the authors’ experience with analysing and deciphering nursing labour market trends within the Canadian context. Note that the multi-disciplinary authorship team has had several decades of research experience in investigating the training, recruitment and retention of nurses, human resources policy analyses, labour economics and human resources management.

### Budget cuts and economic downturns: why are nurses susceptible?

Economic downturns and recessions force governments to restrict their overall public spending. The budget for healthcare programmes, constituting a sizable proportion of the gross domestic product (GDP; e.g., 11% of Canada’s GDP in 2011), is inevitably affected [1,3].

Human resources consume a sizeable proportion of health expenditures with considerable variability in how these workers are paid. This, in turn, means that an important impact of economic downturns is on the ability of governments to maintain and pay for the health workforce. Attempts to decrease healthcare expenditures might also include downsizing of institutions and consequently the size of the workforce. Such a strategy has a strong impact on the nursing workforce for two main reasons. Firstly, nursing is often the largest professional group in the healthcare sector; thus any workforce downsizing attempt will disproportionally affect them. Secondly, an overwhelming majority of nurses are salaried employees in healthcare organisations (as compared to physicians who are self-employed professionals working on a fee-for-a service or capitation basis). Strategies to cope with fiscal pressures may have a direct effect on nurses if institutions opt to balance their budgets by downsizing their nurse workforce or an indirect effect if institutions downsize services and decrease the number of hospital beds [16,17].

### The effect of economic downturns on the nursing labour market

The reviewed literature reports five key indicators measuring the effect of economic downturns on the nursing labour market: demand, supply, salary, turnover and shortages. To facilitate stakeholders’ assimilation of the short-term and long-term effects of economic downturns on the nursing labour market, we propose a simplified framework in Table 1. This framework could serve as a convenient reference guide for nursing and human resources managers, leaders and planners on potential nursing labour force dynamics during and after an economic downturn. It is based on evidence compiled from the literature coupled with the authors’ experience in workforce planning and their analyses of nursing labour market trends.

**Table 1:** Analytical framework on the short-term and strategic impact of economic downturns on the nursing labour market

**Labor market indicator**	**Temporary/ short term**		**Strategic/ long term**	
	**During an economic downturn**	**Potential reasons**	**Post economic downturn**	**Potential reasons**
**Demand for nurses**	**Decrease**	- Downsizing of institutional operations	**Increase**	- Need to recoup lost nursing jobs
		- Budgetary constraints	(Slowly if jobless recovery & fast under full recovery)	- Services restored or expanded
		- Service reductions		- Need to replace retiring baby boomers
**Supply of nurses**	**Increase**	- Nurses increase number of hours worked	**Decrease**	- Decreased enrollment in & graduation from nursing programs at time of recession
		- Inactive nurses return to the labor market		- Decreased labor force participation
		- Multiple job holdings for some		- Increased nursing dropouts from nursing labor market
				- Difficulty in attracting nurses working in other industries or those that sought early retirement
**Nursing Salaries**	Frozen or decreased	- Increased supply coupled with decreased demand	**Increasing**	- Decreased supply & increased demand
				- Need to attract inactive nurses back
**Nursing turnover**	Decreased	- Need for work with decreased family earnings	**Increased**	- More jobs available in the market
		- lack of job vacancies		- decreased need for work withincreased family earning
		- Reduced likelihood that nurses leave their job		- Stress and burnout
**Nursing shortages**	Temporary perception of surpluses rather than shortages	- Disturbed labor market dynamic	**Aggravated**	- Supply–demand imbalances
		- More supply less demand		- Increased population needs
				- Decreased proportion of active nurses
				- Retiring baby boomers

#### Economic downturns and the demand for nurses

Economic downturns and recessions impact the healthcare sector with varying effects on the demand, supply and other labour market indicators for nurses depending on the jurisdiction (Table 1) [2,18]. On the demand side, they are likely to decrease the demand for nurses as well as the need for nursing services. Restricted availability of public and private funds in the healthcare sector might push healthcare organizations to downsize their operations and consequently the size of their nursing workforce [6,7,19].

Recent evidence from the current economic downturns suggests that the health systems in many European countries are contracting or freezing the size of their nursing workforce. For example, despite the estimated shortage of 60,000 nurses in Italy, the Italian government recommended that institutions revisit nursing workforce standards and freeze the recruitment of healthcare workers [20]. Note that younger nurses are disproportionally affected by workforce downsizing since job cuts usually start from the bottom in a unionized environment [21].

Similarly, the Spanish economic crisis has resulted in reductions in the public financing of the healthcare sector which precipitated salary decreases and job freezes in the nursing workforce. This is quite disconcerting since Spain has a lower nurse-to-population ratio compared to other Organization for Economic Cooperation and Development (OECD) countries (7.5 per 1000 population, compared to an average of 9.6 in other OECD countries) [22]. Furthermore, Greece continues to experience a shortage of nurses because of the aging population, the demands of integrating new technology and rising consumer expectations, but has limited ability to meet these needs given its current economic crisis [23].

The need for nursing services might also affect the demand for nurses in other ways. On the one hand, there may be a decrease in occupational injuries due to the contraction in the size of the active workforce. On the other hand, since poverty and unemployment are associated with poorer health, need might increase particularly for those who become unemployed [24-27]. Yet, such demand might be mitigated by the unwillingness of people to pay out of pocket should they lose their insurance coverage [20].

In addition, demand for nurses might be focused on specific sectors/sub-sectors of employment as care shifts from (costly) institutions to the (more affordable) community [28]. The demand for nurses might also be focused on less costly nurses (e.g., practical nurses within the Canadian context) possessing a narrower scope of practice or cheaper non-nursing substitutes (e.g., personal support workers, nursing aides, etc.) [29]. The implications of this skill mix change might extend beyond affecting nurses to affecting the outcomes of patient care [30-32].

#### Economic downturns and the supply of nurses

Economic downturns may cause an unanticipated increase in the supply of nurses in the healthcare market. Decreased family earnings coupled with concerns about job security may push nurses to reintegrate into the labour market and to remain in their current jobs [33]. Thus, increased supply may result from an increased number of inactive nurses returning to the active workforce, increasing the number of working-hours or holding multiple jobs [18,34].

#### Economic downturns and nursing wages

Increased supply and decreased demand for nurses may push nursing wages down over time. Under publically financed healthcare systems and unionized environments, nurses might be shielded from the wage effects of economic downturns in the short-run. However, if economic troubles prevail for longer periods, nurse wages may eventually decline. In addition, if labour agreements curtail the ability to adjust wages downward, the employers may reduce staff numbers via layoff or via service cuts.

This wage effect is currently observed in some countries where the effect of economic downturns is more pronounced. For example, Spain recently passed a law that aims to reduce healthcare spending by introducing a 5% cut in salaries [22]. Similarly, wage decreases have been reported in other troubled economies [22,35,36]. Interestingly, a recent salary survey of nurses in the US revealed a decrease in nurse leaders’ wages at a rate that is comparable to that of their Spanish counterparts (5.2%), but decreases only influenced female workers [35]. Such findings bring to question the influence of economic downturns on the gender equity of wages in a professional workforce.

Such wage decreases might precipitate increased financial burdens on the nursing workforce with an increased number of nurses in financial hardship [37].

#### Economic downturns and nurses’ turnover

Under normal circumstances, nurses dissatisfied with aspects of their work environment would leave their work setting and seek employment elsewhere (or perhaps become unemployed). Economic downturns might prevent this from happening due to the decreased job vacancies and increased financial pressures for families [18]. For example, a recent survey of US nurses reports that the majority of nurses believed they were inappropriately compensated. However, many nurses consider themselves privileged to be still working under a serious economic downturn [35].

#### Economic downturns and nursing shortages

The aforementioned effects of economic downturns on the labour market may provide temporary relief of previously reported nursing shortages. With more nurses rejoining or increasing participation in the nursing labour market, some of the existing nursing vacancies are filled. Similar influences of recessionary trends on nursing shortages were recently reported in the US [2], with increased employment of returning nurses and new nursing graduates [2,38]. Nursing shortages may also decrease because of the cancellation of vacancies due to lack of funds to hire new nurses. Indeed, it is not uncommon to observe hiring freezes in the market during economic downturns [20].

### What happens to the nursing labour market post economic downturns?

The framework outlined in Table 1 highlights some of the anticipated nursing labour market trends in the aftermath of an economic downturn. With respect to the demand for nurses, it is anticipated that the restoration, expansion and improvement of patient care would necessitate the quick hiring of nurses, which would increase the demand for nurses in the market. In practice, the nature of the recovery would dictate what could happen on the demand side of the nursing labour market. Should recovery be jobless, the growth in the demand for nurses will be slow. However, if the recovery leads to job creation, the demand for nurses would increase considerably [39].

On the supply side, the longer-run effect of economic downturns might be a decrease in the supply of nurses. The supply of new nurse graduates decreases due to space constraints in educational institutions, shortages in the number of faculty members, fewer nursing preceptors at healthcare organizations and diminished scholarships available for students [22,40]. For example, recent analyses reveal that budget cutbacks to educational institutions in Ireland reduced the nursing students’ acceptance by up to 1200 in 2009 [41].

Increased turnover is anticipated with improved economic conditions. This also contributes to a decrease in the supply of nurses with decreased labour force participation for some nurses (with increased family earnings), especially those with small children and older nurses who are closer to retirement [3]). Some nurses might also drop out of the labour market due to accumulated stress and burnout precipitated by the heavy workloads and poor work environment during economic downturns [39,42,43]. As mentioned above, economic recovery might also increase the number of jobs available in the healthcare market and other markets.

Reduced supply coupled with increased demand for nurses will eventually push nursing wages up when the economy recovers [21]. Again, wage agreements that are binding for a number of years may delay these wage adjustments and thus impact the ability of institutions to quickly replenish their nursing labour force.

### Implication for policy and planning

Economic downturns place healthcare planners and decision makers under tremendous pressure to balance budgets and cut expenses. Under economic downturns, difficult decisions have to be made in a short period with little opportunity to consult with concerned stakeholders (i.e., patients, nurses, analysts). A clearer understanding of nursing labour market dynamics will help policy makers take decisions that would help them overcome the economic downturn while protecting the strategic interests of the healthcare system.

Despite observing a decrease in the demand for nurses in the labour market during economic downturns, nursing stakeholders should keep in mind that the demand for nurses will eventually increase upon economic recovery. Additional nurses will also need to be hired to address growing population needs, recoup jobs lost during economic downturns, and replace retiring nurses in an aging workforce [44,45]. With respect to the supply of nurses, nursing planners and stakeholders should refrain from eroding the training capacity for nurses during an economic downturn. Such a risky policy option could lead to serious demand–supply imbalances in the nursing labour market in the long-run.

Nursing managers, leaders, planners and policy makers are cautioned against adopting staff layoffs as a strategy to balance their budgets. Layoffs will increase the workload and decrease the morale and productivity of working nurses [8,30,46] and rehiring nurses back will be a costly and time-consuming process [47,48]. Furthermore, the reintegration of inactive nurses into the labour market will necessitate additional investments in a targeted and timely recruitment strategy [49]. A combination of decreased demand and increased layoffs for younger nurses will eventually aggravate the aging workforce problem [50].

At the institutional level, hospitals should prepare for intense recruitment and retention efforts to recover from the job losses caused by the recession, and potentially aggravated by the retirement of baby boomers from the nurse workforce. Avoiding this shortfall requires targeted interventions on the system and institutional levels [2,34]. At the system level, it is essential to maintain adequate funding for nurses training and professional development programs. Institutions should be incentivised to cut expenses through improving efficiency and performance rather than lay off staff. Furthermore, particular attention should be dedicated to the integration of younger nurses into the workforce. One good example is the Nursing Graduate Guarantee in Ontario, Canada. This initiative of the Ontario Ministry of Health and Long-Term Care is intended to ensure that every new nursing graduate will have the opportunity to find a full-time job upon graduation [51].

Interventions at the institutional level may include job-sharing as a way to enhance nurse retention amid budgetary constraints [52]. Other retention initiatives focus on good nursing management during difficult times, including improved nurse recognition, enhanced transparency and communication opportunities, decentralised decision-making, enhanced nurse-physician relationships and improved group cohesion [43,53,54]. Particular attention should be dedicated to providing nurses with professional development opportunities, which have a high impact on retention [55,56].

A number of shortcomings in this manuscript are worth mentioning. First, the literature search supporting the findings in this manuscript might have missed some of the published or grey literature on the topic. Second, some of the reported labour market dynamics in the proposed framework may not be applicable to some jurisdictions or may not account for certain local market specificities. Finally, although some of the discussions and findings of this manuscript are global in nature, most will be more relevant to developed contexts. Note that lack of primary and secondary workforce data might be a barrier to observing workforce trends in developing contexts.

## Conclusions

Healthcare human resources leadership entails seeing beyond the temporary trends by investing in the nursing workforce despite economic turbulence. We argue that this is the optimal means to achieve efficiency and effectiveness, while enhancing patient safety and protecting one of the most valuable assets in a healthcare system–nurses.

Lack of understanding of labour market dynamics and trends might mislead policy makers to make misinformed workforce downsizing decisions that are often difficult and expensive to reverse. Leadership and sound decision making dictates that policy-makers look beyond the temporary relief that might be achieved from staff downsizing decisions and consider the long-term strategic interests of the healthcare system. In the aftermath of an economic downturn, the costs of attracting nurses back often outweigh the short-term cost savings. Effective management would support the nursing workforce by creating attractive and stable work environments to retain nurses at a manageable cost.

## Competing interests

The authors declare that they have no competing interests.

## Authors’ contributions

MA conceptualised the topic, carried out the literature analysis and wrote the first draft of the manuscript. AB helped with the conceptualisation of the topic and reviewed the manuscript providing valuable advice. AL helped conceptualise the labour market implications and thoroughly reviewed the manuscript. RD reviewed and edited the manuscript enhancing its rigor and intellectual content. All authors read and approved the final manuscript.
